# Use and non-adherence to antiretroviral therapy among Refugee HIV positive pregnant mothers aged 18–49 years in Kyangwali Refugee Camp, Western Uganda

**DOI:** 10.21203/rs.3.rs-3961640/v1

**Published:** 2024-02-20

**Authors:** Joan Tusabe, Joanita Nangendo, Michael Muhoozi, Herbert Muyinda

**Affiliations:** Makerere University, College of Health Sciences School of Public Health, Kampala, Uganda; Makerere University, College of Health Sciences, School of Medicine, Clinical Epidemiology Unit, Kampala, Uganda; Makerere University Center for Health and Population Research; Makerere University, College of Health Sciences, Child Health and Development Center

**Keywords:** Antiretroviral therapy, Use, Non-adherence, Refugees, Pregnant mothers

## Abstract

**Introduction:**

Refugee HIV positive mothers experience significant obstacles in accessing, utilizing and adhering to antiretroviral therapy (ART). Identifying ART non-adherence can help enforce interventions aimed at improving adherence and subsequently effectiveness of ART among the refugee mothers. We describe the use and the factors associated with non-adherence to ART among Refugee HIV positive pregnant mothers aged 18–49 years in Kyangwali Refugee Camp, Uganda.

**Methods:**

We conducted a cross-sectional study among HIV positive pregnant mothers aged 18–49 years in Kyangwali refugee camp between May and June 2023. Using a structured questionnaire, we collected data on use, and factors associated with non-adherence to ART. We used modified Poisson regression analysis to determine factors associated with non-adherence to ART.

**Results:**

Of the 380 participants enrolled, 192 (50.5%) were married, mean age 32.1 years. Overall, 98.7; 95% CI [97.5–99.8%] were using ART and 27.4; 95% CI [22.9–31.9%] were non-adherent. Non-adherence was associated with; Initiating PMTCT care in the third trimester of pregnancy (aPR: 2.06; 95% CI: 1.27–3.35), no need to get permission to seek PMTCT services aPR 1.61; 95% CI [1.07–2.42] and poor attitude of PMTCT providers aPR 1.90; 95% CI [1.20–3.01].

**Conclusion and recommendations:**

Non-adherence to ART was generally high; therefore limiting the effectiveness of the PMTCT program in this setting. Refugee context specific education interventional programs aimed at early initiation into HIV care, strong social and psychological support from families, communities and health care providers are vital to improve adherence this setting.

## Introduction

Globally, HIV remains a major global public health concern with especially countries in Sub-Saharan Africa (SSA) reporting increasing trends in new infections [1]. As of 2022, there was an estimated 39.0 million people living with HIV, with two thirds in SSA alone [1]. In Uganda, prevalence of HIV among adults in the general population is at 5.8% but higher among women at 7.2% [2]. In the refugee settlements, prevalence of HIV among adults is 1.8% among women and 1.1% among men [3]. Refugees are at an increased risk of contracting HIV due to the extended displacement and associated disruption to their lives [4]. They are often accused of importing HIV to the host communities, and are therefore discriminated against [4] and their prioritize are characterized by day-to-day survival such as finding food, safety, and shelter over health [4–6]. In such circumstances, women often engage in commercial sex for food, shelter, clothing and other basic commodities [4], which puts them at a high risk of acquiring HIV and subsequently risks of vertical transmission of the infection to their unborn babies and breast-feeding infants[4].

Prevention of Mother to Child Transmission (PMTCT) is a proven HIV prevention intervention set and adopted by various health systems globally [7]. PMTCT programs provide a range of services to mothers and their infants. These services include HIV testing and counselling, ART initiation, safe sex practices, safe child birth practices, appropriate feeding practices and viral load testing and ART prevention for exposed infants [8] [9]. In order to achieve viral load suppression and prevention of mother to child transmission, ART should be initiated early in pregnancy and optimal adherence observed [10, 11]. However, evidence shows various gaps in implementation of PMTCT programs. In a systematic assessment in low and middle-income countries, nearly half of HIV positive expectant mothers neither received ART prophylaxis during antenatal care (ANC) nor delivered in health facilities [12]. A study in Northern Uganda using health facility data from 2002 to 2011 showed that only 69.4% of HIV positive gravid mothers were started on ART for prophylaxis [12]. Mukose et al in a study conducted in Central Uganda found that 91% of mothers received a prescription of ART; of whom 93.3% started swallowing their medicines and only 76.8% achieved optimal adherence to ART [11]. This study describes the use and the factors associated with non-adherence to ART among Refugee HIV positive pregnant mothers aged 18–49 years in Kyangwali Refugee Camp, Western Uganda using the social ecological model. We observe that although PMTCT services exist, refugee pregnant mothers experience significant obstacles in accessing, utilizing and adhering to such services which negatively affects the effectiveness of the program. Refugee context specific education interventional programs aimed at early initiation into HIV care, strong social and psychological support from families, communities and health care providers are vital to improve adherence this setting.

## Materials and Methods

### Study design

We conducted a cross-sectional study employing a quantitative methods among refugee HIV positive pregnant mothers aged 18–49 years in Kyangwali refugee camp. This study adopted and modified the social ecological model (SEM) [13] to study factors associated with non-adherence to lifelong ART among the refugee HIV positive pregnant mothers. The SEM puts into consideration the individual, and their relations to peers, family, community of residence and organization. It therefore considers five levels that is individual/intrapersonal, interpersonal, community, organization and public policy [13]. However, we focus only on the first four levels and did not study any factors related to public policy in this work.

### Study setting

This study was conducted between May and June 2023 among refugee HIV positive pregnant mothers aged 18–49 years in Kyangwali refugee camp, Western Uganda. Kyangwali refugee camp lies in Kikuube district in Western Uganda, along Lake Albert at the border between the Democratic Republic of Congo and Uganda. The refugee settlement covers over 90 square kilometres and is divided into 14 villages consisting of 10 to 20 housing blocks each [14]. Top administration of the camp is managed by the Office of the Prime Minister (OPM) which retains responsibility concerning; on-site settlement management teams and selection of Refugee Welfare Councils. Given its proximity to Eastern Congo, majority of the population is Congolese [14], and as of January 2021 the settlement had over 125,039 people in 42,428 households. Of these, 81% are women and children, and 19% are youth aged between 15–24 years [15].

### Study population and sampling

A pregnant woman was eligible for the study if she: 1) was aged 18–49 years, 2) had available HIV/PMTCT records at a health facility, and 3) was willing to consent to join the study. An individual was excluded if she: 1) could not speak English, Runyoro or Swahili, and 2) was found admitted at a health facility for any medical condition at the time of the study.

We estimated the sample size of 380 pregnant mothers using both the Leslie Kish and finite population formulae [16, 17]. This was done under the following assumptions: 55% uptake of PMTCTC [12] among pregnant mothers a 5% level of precision and 95% confidence interval. Participants were attached to six health facilities of Kasonga H/C III, Maratatu H/C III, Rwenyawawa H/C III, Kansonga H/C IV, Kyangwali H/C IV, and Rwenyawawa H/C IV. For purposes of representativeness, we employed simple random sampling using computer-generated random numbers and proportion to size sampling for each of the health facilities.

### Data collection tool, measurements and procedure

Data were collected using a structured questionnaire administered in face-to-face interviews. The questionnaire included questions on socio-demographic characteristics, knowledge and attitudes about HIV and PMTC, family/partner, community and organizational related factors, current use and adherence to lifelong ART, motivation to continue using ART and reasons for non-use and non-adherence to ART. Use and non-adherence to lifelong ART were the dependent variables and were measured by self-reports and verified with drug record availability at the respective health facility reported by the participant. Use was defined as having received a prescription, starting to swallow ART and currently swallowing ART while non-adherence was defined as taking less than 95% of the ART doses (≤ 29 doses) in the 30-days before the interview month [11]. Non-adherence was assessed through self-report by asking for the number of ART doses taken in the past 30 days. The study team included the first author (JT), two counsellors, one community linkage facilitator, one Village health team (VHT) team and two research assistants (RAs). All the research team members were experienced in community research work, spoke and read English, Runyoro and Kiswahili. The counsellors had a diploma level of training in Community HIV/AIDS care and management, the RAs had a degree-level training in Environmental Health Sciences, while the community linkage person and VHT had lower secondary school of education. Prior to the start of data collection, RAs were trained for four days on all study procedures to clarify their responsibilities and pre-test the study questionnaire. The counsellors helped in identifying registered pregnant mothers on the PMTCT program from the facility records while the community linkage facilitator and VHT helped in identifying the selected mothers from the refugee camp. The RAs individually administered questionnaires to each participant. At the end of each day, all collected data were reviewed for accuracy and completeness by the study PI (JT).

### Statistical analysis

We summarized continuous variables using mean and standard deviation, and used frequencies and percentages for the categorical variables. We defined the outcome (non-adherence to lifelong ART) as taking less than 95% of the ART doses (≥ 29 doses) in the 30-days before the interview month [11] which we measured on a binary scale as a proportion. We used modified Poisson regression analysis to assess for factors associated with non-adherence to PMTCT and measured associations as prevalence ratios (PR) and their 95% CI. At bivariate analysis, we considered variables with *P* < 0.20 as significant for multivariate analysis. We also added to the multivariable model variables reported as confounders in literature even if they were not significant at bivariate analysis. Modified Poisson regression model was used because data were cross-sectional and PR were more conservative in magnitude than prevalence odds ratios (POR) for a relatively common outcome, ie, > 10% [18, 19]. We checked for correlation and multi-collinearity by conducting a correlation coefficient matrix and a variance inflation factor (VIF) test. We conducted likelihood ratio tests to identify the best fitting model. Adjusted prevalence ratios with *P*-value < 0.05 were considered statistically significant.

## Results

### Characteristics of the study population

We enrolled 380 participants into the study. Participant had a mean age of 32.1 years, 51.8% were married, 50.3% had attained primary education and 69.5% relied on farming as an occupation. 64.0% had disclosed their HIV status to the partner or family member and 34.2% needed permission from partner or family member to seek for PMTCT services (Table 1).

Only 19.7% belonged to a community support group, majority (98.2%) did their initial HIV testing from the health facility, 26.8% did not receive any counselling before the HIV test and 1.6% did not receive any counseling post the HIV test. More than half (68.7%) mentioned that the transport costs to PMTCT health facilities were not affordable. (Table 2).

Although most (99%) participants were using ART, 27.4% did not adhere to ART. About 99.2% intended to deliver from a health facility and 98.7% to continue using ART in the future (Table 3).

At multivariable analysis, non-adherence was associated with one factor under each level of the SEM. The factors were duration of pregnancy at PMTCT initiation, need for permission from partner or family member and attitude of the PMTCT service providers.

Non –adherence to ART was associated with initiating into PMTCT care in the third trimester of pregnancy; aPR 2.06; 95% CI [1.27–3.35], no need to get permission from either partner or family member; aPR1.61; 95% CI [1.07–2.42] and perceived poor attitude of PMCTC service providers; aPR 1.90; 95% CI [1.20–3.01]. Table 4

## Discussion

This study assessed the current use and the factors associated with non-adherence to lifelong ART among Refugee HIV positive pregnant mothers aged 18–49 years in Kyangwali Refugee Camp, Western Uganda. The study findings indicate that 98.7% of the pregnant mothers were currently using lifelong ART, 99.2% intended to deliver from the health facility and 98.7% intended to continue using lifelong ART in the future. However, non-adherence to lifelong ART was quite high at 27.4%; and was significantly associated with initiating PMTCT care in the third trimester of pregnancy, no need for permission to seek for PMTCT care and perceived poor attitude of PMTCT service providers.

Our finding of current use of lifelong ART among pregnant mothers aged 18–49 years at 98.7% is higher than the finding of the Uganda Refugee Population-Based HIV Impact Assessment (RUPHIA) 2021 survey of 87.0%. [3]. The observed difference can be attributed to two factors. Firstly, while the RUPHIA survey was community based and among all refugee settings in Uganda, our study was both facility and community based. It is more likely that participants identified from facility records would be found using ART at the moment. Secondly, while the RUPHIA survey was conducted among the general population, our study was conducted among a special population of pregnant mothers. In comparison to the general population, evidence indicates that the desire to have HIV free infants is likely to positively influence pregnant mothers to use lifelong ART [11]. Similarly, our finding of current use of lifelong ART is higher than reported in other studies conducted among pregnant mothers in non-refugee settings. [20, 21].

The high levels of current use and intention to continue using lifelong ART are promising. However, the observed non-adherence level of 27.4% is worrying since the effectiveness of the PTMTC programs directly depends on the mother’s adherence to lifelong ART [10, 11]. The non-adherence observed in our current study is slightly above what Mukose et al found in a study conducted among pregnant and lactating mothers in Central Uganda [11]. This can partly be explained by the use of a combination of modern health facility and traditional health services among refugees. The use of both services occurs even when there are better health services in stable refugee settings [22]. It is therefore important to build capacities for both modern health facility and traditional health personnel in partnership. More so, such built capacities need to pay attention to education and counselling strategies regarding the importance of adherence to lifelong ART.

Our study findings also indicated three factors associated with non-adherence with one factor under each of the three levels of the SEM.

At the intrapersonal/individual level, we found that mothers who initiated their life long ART/PMTCT care in the third trimester of pregnancy were more likely to be victims of non-adherence. This finding is in line with other previous studies [23, 24]. Literature indicates that late presentation into HIV care poses a higher cumulative risk of HIV transmission to others, less chances of responding to treatment, non-adherence and increased financial strain on health services systems [25, 26]. Similarly, starting PMTCT during the third trimester has significant consequences to both the mothers and the unborn babies. Firstly, the available short time discourages the mother from adhering to achieve viral suppression and reduce the risk of transmitting HIV to their child. Secondly, there is no ample time needed to adapt to a new medication routine, deal with the drug related side effects and other psychological aspects that come with the pregnancy and the new HIV diagnosis. It is therefore vital to develop refugee context specific education interventional programs aimed at providing knowledge on the available health care and HIV specific services in the refugee camp to improve the timely consumption of such services. There is also need for assessing other reasons for late utilization of ANC/PMTCT and other related services beyond the individual level.

At the interpersonal level, we found that mothers who did not need to obtain permission from a partner or family member to seek for PMTCT services were more likely to be victims of non-adherence. This finding may seem contradictory, however, the need to obtain permission from a partner or family member in the refugee context may indirectly imply availability of a family support system that not only provides permission to seek for PMTCT services but also social, psychological and financial support to the mother. While family support plays a significant role in ART adherence [27–29], evidence indicates that refugee communities usually lack such support due to family separation. Concerns about the need to flee quickly without family, separation in the process of displacement, family members going missing, or substantive barriers to family reunification following safe resettlement in a host country have been reported among refugee communities [30, 31]. Mechanisms to limit family separation during resettling of refugees and creation of strong social and psychological support systems in settlement areas are crucial.

Lastly, at organization level, mothers who perceived the attitude of PMTCT service providers as being poor were more likely to non-adhere to lifelong ART. Previous studies have illustrated how health workers’ poor attitudes and non-professional behaviour could impinge on clients’ adherence to medications. They have also demonstrated the need to provide good working environments and motivation strategies to health care providers to effectively provide services [32–34].

### Study limitation

The main limitation of this study is the fact that data was collected by self-report. This could have been subject to recall bias and social desirability bias. However, we limited our recall period to the last thirty days and interviews were conducted maintaining utmost confidentiality and in a conducive environment for participants to provide an honest view.

## Conclusion

Non-adherence to ART was generally high; therefore limiting the effectiveness of the PMTCT program in this refugee setting. Duration of pregnancy at PMTCT initiation, need for permission from partner or family member and perceived attitude of the PMTCT service providers influenced non-adherence. Refugee context specific education interventional programs, strong social and psychological support systems both from family community and health facilities are vital to improve adherence. Assessing other reasons for late utilization of ANC/PMTCT and other related services beyond the individual level is also needed.

## Figures and Tables

**Table 1:** Characteristics of the participants enrolled in the PMTCT study in Kyangwali Refugee camp, Western Uganda, May 2022 (n=380)

Characteristics	Frequency (Percentage)
	(n=380)
Mean age: 32.1 years (SD: 6.0 years)	
**Age groups**	
18–24 years	43 (11.3)
25–34 years	192(50.5)
35–49 years	145(38.2)
**Marital status**	
Single (Not in union)	197 (51.8)
Married( In union)	183 (48.2)
**Number of living children**	
No children	9 (2.4)
1–2 children	88 (23.2)
3–5 children	196 (51.6)
6 children and above	87 (22.9)
**Education level**	
None	152 (40.0)
Primary education	191 (50.3)
Secondary education and above	37 (9.7)
**Religion**	
Catholic	154 (40.5)
Anglican	80 (21.1)
Muslim	35(9.2)
Others	111 (29.2)
**Occupation**	
Farming	264 (69.5)
Business (sales and services)	55 (14.5)
Manual labour	16 (4.2)
Formal employee	12 (3.2)
None	23 (8.7)
**Partner’s level of education (n=190)**	
None	41 (21.6)
Primary education	108 (56.8)
Secondary education and above	41 (21.6)
**Partner’s occupation (n=190)**	
Farming	98 (51.6)
Business (sales and services)	23 (12.1)
Manual labour	40 (21.1)
Formal employee	12 (6.3)
None	17 (8.9)
**Duration of pregnancy at first ANC/PMCTC visit**	
First trimester (1–3 months)	302 (79.5)
Second trimester (4–6 months)	63 (16.6)
Third trimester (7–9 months)	15 (3.9)
**Need permission to go for PMTCT services**	
Yes (needs)	164 (34.2)
No (doesn’t need)	216 (56.8)
**Disclosed HIV status to partner/family member**	
Yes (Disclosed)	243 (64.0)
No (Hasn’t disclosed)	137 (36.0)

**Table 2:** Community and institutional level characteristics of participants enrolled in the PMTCT study in Kyangwali Refugee camp, Western Uganda, May 2022 (N=380)

**Belong to any community support group**	
Yes	75 (19.7)
No	305 (80.3)
**Facility level attended**	
H/C II	9 (2.4)
H/C III	192 (50.5)
H/C IV	179 (47.1)
**Place of initial HIV testing**	
Health facility	373 (98.2)
Community linkage point	7 (1.8)
**Mode of pre-HIV test counselling**	
Individual	197 (51.8)
Group	81 (21.3)
Not done	102 (26.8)
**Mode of post HIV test counselling done**	
Individual	356 (93.7)
Group	18 (4.7)
Not done	6 (1.6)
**Follow up counselling done**	
Yes	250 (65.8)
No	130 (34.2)
**Cost of transport to health facility**	
Affordable	119 (31.3)
Not affordable	261 (68.7)
**Attitude of PMTCT service providers**	
Good	361 (95.0)
Poor	19 (5.0)

**Table 3:** Use and non-adherence to ART among participants enrolled in the PMTCT study in Kyangwali Refugee camp, Western Uganda, May 2022 (N=380)

Variable	Measure
**Outcome variable**	
Currently taking lifelong ART (Yes), n (%; 95%CI)	375 (98.7 ) [97.5–99.8]
Adhered to lifelong ART in the last one month (Yes), n (%; 95%CI)	276 (72.6) [68.1–77.1]
Didn’t adhere to lifelong ART in the last one month	104 (27.4) [22.9–31.9]
Intended to deliver at health facility	377 (99.2)
Plan to continue on lifelong ART/ PMTC services in the future (Yes), n (%)	375 (98.7)
**Other variables on PMTCT and HIV transmission**	
Heard of PMTCT services (Yes), n (%)	355 (93.4)
Believed that HIV can be transmitted from mother to baby during pregnancy (Yes), n (%)	320 (84.2)
Believed that HIV can be transmitted from mother to baby during delivery (Yes), n (%)	351 (92.4)
Believed that HIV can be transmitted from mother to baby during breastfeeding (Yes), n (%)	353 (93.0)
Believed that an HIV pregnant mother can still look health: (Yes), n (%)	319 (84.0)
Believed that every pregnant mother should be screened for HIV (Yes), n (%)	371 (97.6)
Believed that every HIV positive pregnant woman should be on ART (Yes), n (%)	371 (97.6)

**Table 4:** Factors associated with non-adherence to lifelong ART among participants enrolled in the PMTCT study in Kyangwali Refugee camp, Western Uganda, May 2022 (n=380)

Factors	Non-adherence to ART n=104 n (%)	Crude Prevalence ratios (95% CI)	P values	Adjusted prevalence ratios (95% c=CI)	P values
**Age**					
18–24 years	17 (16.4)	1			
25–34 years	44 (42.3)	0.58 [0.37–0.91]	0.018	0.67 [0.42–1.10]	0.081
35–49 years	43 (41.3)	0.75 [0.48–1.17]	0.208	0.79 [0.51–1.22]	0.285
**Marital status**					
Married( In union)	44 (42.3)	1.0		1.0	
Unmarried (Not in union)	60 (57.7)	0.79 [0.57–1.10]	0.164	0.77 [0.44–1.35]	0.364
**Number of living children**					
No children	2 (2.0)	1.0			
1–2 children	31 (29.8)	1.59 [0.45–5.57]	0.472		
3–5 children	46 (44.2)	1.06 [0.30–3.69]	0.932		
6 children and above	25 (24.0)	1.29 [0.36–4.60]	0.691		
**Religion**					
Catholic	51 (49.0)	1.0		1.0	
Anglican	20 (19.2)	0.75 [0.49–1.17]	0.212	0.77 [0.49–1.21]	0.262
Muslim	7 (6.7)	0.60 [0.30–1.22]	0.158	0.59 [0.30–1.16]	0.123
Others	26 (25.0)	0.71 [0.47–1.06]	0.094	0.73 [0.48–1.09]	0.118
**Education level**					
None	41 (39.4)	1.0			
Primary level	51 (49.0)	0.99 [0.70–1.41]	0.955		
Secondary level and above	12 (11.5)	1.20 [0.70–2.05]	0.499		
**Duration of pregnancy at PMTCT initiation**					
First trimester	72 (69.2)	1.0		1.0	
Second trimester	22 (21.2)	1.46 [0.99–2.17]	0.057	1.31 [0.89–1.95]	0.171
Third trimester	10 (9.6)	2.80 [1.85–4.21]	0.000	2.06 [1.27–3.35]	**0.004**
**Partner’s level of education**					
None	10 (22.2)	1.0			
Primary level	27 (60.0)	1.03 [0.54–1.93]	0.939		
Secondary level and above	8 (17.8)	0.80 [0.35–1.83]	0.596		
**Disclosure to partner/family about HIV status.**					
Disclosed	62 (59.6)	1.0		1.0	
Didn’t disclose	42 940.4)	1.20 [0.86–1.67]	0.278	0.86 [0.56–1.32]	0.489
**Require/need permission to pick ART**					
Yes	34 (32.7)	1.0		1.0	**0.022**
No	70 (67.3)	1.56 [1.09–2.23]	0.014	1.61 [1.07–2.42]	
**Belong to community support groups**					
Yes	22 (21.2)	1.0		1.0	
No	82 (78.9)	0.92 [0.62–1.36]	0.667	1.01 [0.66–1.53]	0.974
**Health facility level attached**					
H/C II	3 (2.9)	1.0			
H/C III	44 (42.3)	0.68 [0.26–1.80]	0.445		
H/C IV	57 (54.8)	0.96 [0.37–2.47]	0.925		
**Place of initial HIV testing and counselling**					
Health facility	100 (96.1)	1.0		1.0	
Community	4 (3.9)	2.13 [1.10–4.14]	0.025	1.27 [0.64–2.51]	0.488
**Follow up counselling done**					
Yes	61 (58.6)	1.0		1.0	
No	43 (41.4)	1.36 [0.98–1.88]	0.069	1.17 [0.84–1.62]	0.358
**Perceived cost of transport to health facility**					
Affordable	33 (31.7)	1.0		1.0	
Not affordable	71 (68.3)	0.98 [0.69–1.39]	0.915	0.97 [0.67–1.38]	0.848
**Perceived attitude of the health workers**					
Good	92 (88.5)	1.0		1.0	
Poor	12 (11.5)	2.48 [1.68–3.65]	0.000	1.90 [1.20–3.01]	**0.007**

## Data Availability

All data supporting the results and conclusions of this manuscript are fully available without restriction and are included within the manuscript and its supporting information files.
